# Phenotypic selection of high-yielding, water-productive wheat (*Triticum aestivum* L.) putative mutants under well-watered and limited-irrigation conditions

**DOI:** 10.1038/s41598-026-65822-9

**Published:** 2026-08-13

**Authors:** Ayman Anter Saber

**Affiliations:** Department of Field Crops Research, Biological and Agricultural Institute, National Research Center, Giza, Egypt

**Keywords:** Bread wheat, Mutagen, Mutant lines, Grain yield, Limited irrigation, Water productivity, Genetics, Physiology, Plant sciences

## Abstract

Developing high-yielding and water-stress-tolerant bread wheat cultivars with high water productivity is vital for sustainable wheat cultivation under the limited water resources in Egypt. In this study, bread wheat mutant lines from the M3, M4, and M5 generations and their mother cultivars were evaluated under both well-watered and limited-irrigation conditions over three growing seasons (2022–2024). Eight agronomic traits and water productivity were estimated to detect high-yielding, water stress-tolerant wheat lines with improved water productivity. Significant differences (*p* ≤ 0.05) were observed among the mutant lines for the studied traits under both conditions. Twelve selected mutant lines surpassed their mother cultivars. Mutant line G5 exhibited the shortest plant height with high potential yield, representing a dwarf phenotype. Mutant lines G10, G1, G2 and G4 exhibited the highest actual genetic gain relative to the highest-yielding mother cultivar. High broad-sense heritability coupled with moderate to high genetic advance for the studied traits indicated the predominance of additive gene action and confirmed the effectiveness of phenotypic selection. Drought tolerance indices selected the same promising lines under limited-irrigation conditions. GGE biplot analysis revealed that mutant lines G2, G3, G4, G5 and G6 were superior high-yielding lines, whereas G4, G7, G10 and G11 exhibited greater stability across irrigation regimes. Selected mutant lines exhibited improved water productivity compared with their mother cultivar under both conditions. Selected putative mutant lines will be advanced to multi-environment trials to further evaluate genotype x environment interactions and genetic stability. These mutant lines could serve as valuable genetic resources for bread wheat breeding programs aimed at enhancing grain yield under water-limited conditions.

## Introduction

Bread wheat (*Triticum aestivum* L.) is one of the most important staple crops in Egypt, providing a significant proportion of the calories and protein^1,2^. In addition, wheat straw is also used as a main source of livestock feed^3,4^. However, Egypt imports approximately 60% of its wheat requirements because of rapid population growth and limited arable land^5,6^. The Nile River is the main source of irrigation water, supplying more than 90% of the irrigation water^7^. However, this water resource is no longer sufficient to sustain or maintain agricultural expansion. In addition, climate change is projected to enhance wheat water consumption by approximately 11.0% in northern and middle Egypt^8^. Therefore, developing high-yielding and water-stress-tolerant wheat cultivars has emerged as an essential strategy^9^. Unfortunately, most current cultivars have not been developed to withstand water stress or to achieve high water productivity. Additionally, tolerance to water stress is a complex polygenic trait, strongly influenced by genotype X environmental interactions, which often limit breeding progress^10^. The current genetic diversity for water-stress tolerance in bread wheat remains relatively limited.

Water productivity (WP) represents a fundamental selection criterion for detecting wheat cultivars tolerant to limited-irrigation conditions, as it reflects the efficiency with which plants convert available water into biomass and final grain yield^11,12^. Therefore, enhancing water productivity while boosting productivity has become a major objective in bread wheat breeding programs aimed at developing new cultivars better adapted to water–stress environments^13^.

Mutation breeding is one of the most effective approaches for enhancing genetic diversity in bread wheat^14,15^, owing to the species’ hexaploid nature (2n = 6x = 42, AABBDD), which provides a buffering effect against lethal mutations^16^. Mutagens act upon the entire genome, supporting the modification of key grain yield components in a desirable direction. Improvements in final yield are typically attributed to enhancements in spikes m^−2^, grains per spike, spike yield and spike length^17–20^.

Following the generation of genetic variability, it is necessary to estimate the relative contributions of genetic and environmental factors to the observed phenotypic variation. Several statistical parameters, including the phenotypic coefficient of variance (PCV), the genetic coefficient of variance (GCV), broad-sense heritability, and genetic advance, are commonly employed to enhance selection efficiency and genetic improvement. High heritability (> 60%) coupled with high genetic advance (> 20%) indicates additive gene action, suggesting the potential effectiveness of direct selection^21–23^. Correlation coefficients provide an effective tool for detecting traits associated with final grain yield under different conditions. Grain yield is strongly associated with grain yield components, such as spikes m^−2^, grains per spike, and 1000-grain weight. In addition, other yield-related traits, including plant height and spike length, contribute to improving grain yield. Consequently, direct selection of these traits may lead to substantial yield gains^23,24^.

Drought tolerance indices are commonly applied to assess genotype stability under both non-stress and drought-stress conditions. The Yield Stability Index (YSI) was proposed by Bouslama and Schapaugh^25^, while Fernandez^26^ developed the Stress Tolerance Index (STI) to detect genotypes with superior performance under drought stress conditions. Similarly, Gavuzzi et al.^27^ developed the Yield Index (YI) to assess a genotype performance under water-limited conditions relative to the mean yield. Nevertheless, developing cultivars tolerant to limited irrigation remains a formidable challenge for breeders, due to the multifaceted nature of drought stress and the polygenic inheritance (multiple QTLs) of tolerance traits, which are influenced by environmental interactions^28,29^.

The GGE biplot, based on principal component analysis (PCA), is an effective method for comprehensively exploring multi-environment trial data. It is helpful in summarizing data through the graphical representation of the first two principal components of the multiplicative model^30,31^. Furthermore, the biplot is a proper visualization technique for detecting similarity or dissimilarity among genotypes and is commonly used to select high-yielding and stable genotypes under different conditions^32^.

In this study, phenotypic selection was applied to detect promising mutant lines with desirable and heritable morphological traits across irrigation regimes utilizing multivariate analysis. Therefore, the objectives of this study were to evaluate advanced bread wheat mutant lines across M3, M4 and M5 generations under well-watered and limited-irrigation conditions and to detect high-yielding, water stress-tolerant wheat lines with improved water productivity using multiple selection criteria.

## Materials and methods

### Experiment site

Field experiments were conducted during three consecutive winter growth seasons (2022–2024) at Behbeesheen Village, Beni Suef Governorate, Egypt (29.0667° N, 31.1000° E). This location was characterized by low annual rainfall, elevating the risk of water stress on agricultural production under projected climate change scenarios^33^. Soil physical properties included field capacity (40%), permanent wilting point (20%), and available water capacity (20%, calculated as FC − PWP). Soil bulk density was 1.3 g/cm^− 3^ and the infiltration rate was 1.0 cm h^−1^. Chemical properties included pH (8.5), the soil electrical conductivity (EC) was 2.1 dS m^−1^, organic matter (1.5%) and Ca Co_3_ (8.0%), available N (45 mg kg^−1^), available P (8 mg kg^−1^) and available K (320 mg kg^−1^). Soil physical properties were analyzed according to the procedure described by Carter and Gregorich^34^. Guard rows were planted for each mutant line in each replicate to minimize border effects. Surface irrigation was applied, and all agronomic practices followed the guidelines of the Egyptian Ministry of Agriculture.

### Genetic material (mother cultivars)

This study was part of an ongoing bread wheat (*Triticum aestivum* L.) mutation breeding program aimed at developing high-yielding mutant lines with improved tolerance to limited-irrigation conditions. The earlier generations (M1 and M2 generation) used in this study were described in a previous publication^35^. The pedigree of the mother cultivars were as follows:

Sakha 93 (P1): Sakha 92/TR 810328 S 8871-1S-2S-1S-0S. Sids 13 (P2): KAUZ″S”//TSI//TSI/SNB″S″ICW94-0375-4AP-2AP-030AP-0APS-3AP-0APS-050AP-0AP-0SD. Giza 168 (P3): MIL/BUC//Seri CM93046-8 M-0Y-0 M-2Y-0B. Gemmeiza-9 (P4): Ald”S”/Huac”S”//CMH74A.630/5x CGM4583-5GM-1GM-0GM. Maryout 5 (P5): Giza 162 // Bchʼs /4/ PI-ICW 79Su511Mr-38Mr-1Mr-0Mr.

### Irradiation treatments

At the initiation of the breeding program, 300 dry and healthy seeds per treatment for each cultivar were used. Seed moisture content was adjusted to 12% (w/w) before irradiation to minimize physical damage and enhanced mutation frequency. Seeds of each cultivar were irradiated with three doses of 100, 200, and 300 Gy of gamma rays (Cobalt-60) at the Egyptian Atomic Energy Authority, using a dose rate of 0.9 Gy s^−1^. The corresponding exposure times were 111, 222, and 333 s, respectively. After irradiation, seeds were stored under cool and dry conditions (10 °C) and 40% relative humidity to avoid physiological damage until sowing. The irradiation doses were selected according to previous studies by Naggar et al.^36^ and Aly et al.^37^. In the present study, the plant materials evaluated included advanced mutant lines in the M3, M4 and M5 generations. In the M3 generation, mutant lines were evaluated under well-watered conditions only, while the M4 and M5 generations were evaluated under both well-watered and limited-irrigation conditions. Five mother cultivars were used as controls in all evaluations. Selection was postponed until the M3 generation because bread wheat possesses a hexaploid genome (AABBDD), in which mutant alleles may remain masked during the M1and M2 generations, while greater homozygosity expression is more achieved in the M3 generation, making phenotypic selection more reliable^38^.

### Irrigation regimes

**Fig. 1:**
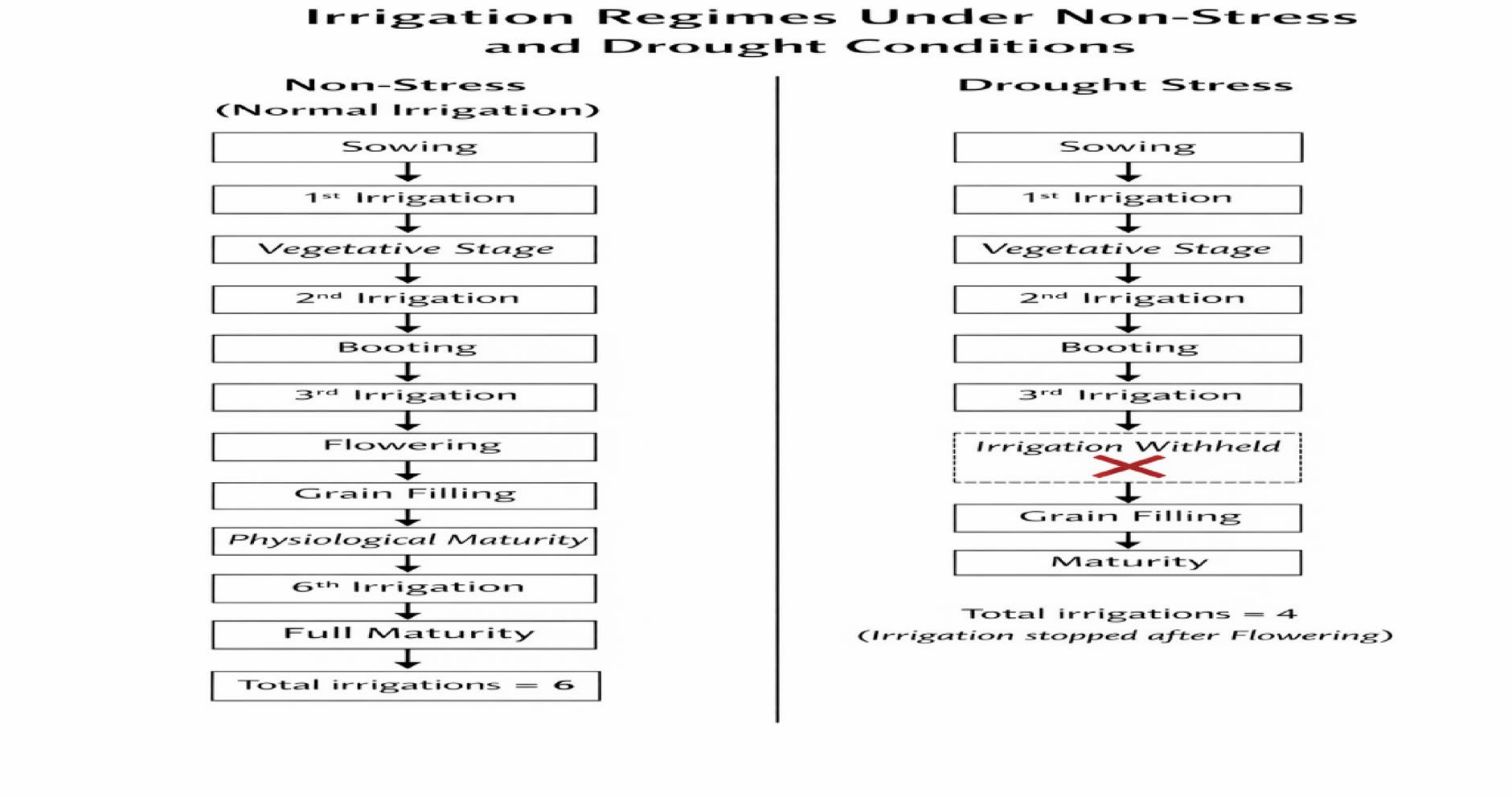
Irrigation regimes applied under well-watered and limited-irrigation conditions.

This study was conducted under current irrigation systems used in Egypt for the following reasons:

1. Breeding wheat for tolerance to limited irrigation has limited progress when selection and evaluation were conducted under artificial conditions that do not reflect actual field water regimes, which reduces the effectiveness of selection^39^.
2. In Egypt, most agricultural lands were irrigated using surface irrigation, where water application was based on irrigation frequency rather than quantified water volume^40^, reflecting the irrigation system under Egyptian conditions. Therefore, in the current study, irrigation treatments were imposed on the number of irrigation events rather than water volume. This tool enabled the evaluation and selection of superior wheat mutant lines under realistic field conditions and water-saving irrigation. Therefore, two irrigation treatments were used to generate environmental variation. The first environment represented well-watered conditions, where irrigation was continued until physiological maturity (farmers practices), resulting in six irrigation events. The second environment represented limited-irrigation conditions (60–65%), where irrigation was continued until flowering stage, resulting in four irrigation events. The 2-irrigation saved approximately 2.245 m^3^ ha^−1^ referenced against modeled requirements of 6738 m^3^ ha^−1^ for this region^7^. These irrigation environments were used as selection pressure to detect mutant lines with high potential yield under both well-watered and limited-irrigation conditions. As shown in Fig. 1, irrigation regimes corresponded to well-watered (100% ETc) and water-limited (~ 60–65% ETc) conditions.

### Selection protocol

In the M3 generation (2022), 40 mutant lines were cultivated alongside their mother cultivars. Based on agronomic performance, 20 promising lines were selected and advanced to obtain the M4 generation (2023). From these, 12 promising mutant lines were further selected and advanced to obtain the M5 generation (2024). The intensity selection (ca. 50%) was coupled with trait differentiation and differential responses of mutant lines under both conditions.

### Experimental layout

Each experimental plot consisted of five rows, 2 m in length and 1 m in width, spaced 10 cm apart, produced a total plot area for each genotype was 6 m^2^. Sowing was done manually in November at a seeding rate of 350 grains m^2^.

### Studied traits

The studied agronomic traits included plant height (PH, cm), spike length (SL, cm), spike weight (SW, g), spike yield (SY, g), grains per spike (GS), 1000-grain weight (GWT, g), spikes m^−2^ (SN), grain yield (GY, t ha^−1^).

### Drought tolerance indices

1. ${\mathrm{MP}} = \frac{{{\mathrm{Yp}}\: + \:{\mathrm{Ys}}}}{2}$. The high value of MP indicated higher productivity^41^.
2. Geometric Mean productivity (GMP) = $\sqrt {({\mathrm{Yp}} \times {\mathrm{Ys}})}$. The high value of GMP related to high yielding genotypes in both well-watered and limited-irrigation conditions^26^.
3. Harmonic Mean (HM) =$\frac{{2({\mathrm{Yp}} \times {\mathrm{Ys}})}}{{(Yp + Ys)}}$. The high HM value indicated that they were more tolerant^42^.
4. Tolerance Index (TOL) = Yp – Ys^43^.A high value of TOL indicates susceptibility of a given genotype^26^.
5. Stress Tolerance Index: (STI)=$\frac{{({\mathrm{Yp}} \times {\mathrm{Ys}})}}{{\left( {\overline{{\mathrm{Y}}} {\mathrm{p}}} \right)^{2} }}$. The high value of STI indicated high yield under well-watered and limited irrigation-conditions^26^.
6. Stress Susceptibility Index (SSI) =$\frac{{[1 - ({\mathrm{Ys/Yp}})]}}{{[1 - \left( {\overline{{\mathrm{Y}}} {\mathrm{s/}}\overline{{\mathrm{Y}}} {\mathrm{p}}} \right)]}}$. Low value indicated more tolerance to limited-irrigation conditions^45^.
7. Yield Index (YI) = $\frac{{{\mathrm{Ys}}}}{{\overline{{\mathrm{Y}}} {\mathrm{s}}}}$. The high value of YI is suitable for limited-irrigation conditions^27^.
8. YSI = $\frac{{{\mathrm{Ys}}}}{{{\mathrm{Yp}}}}$. The high value of YSI indicated stability of the genotype under well-watered and limited-irrigation conditions^43^.

where: Yp = yield of the genotype under non-stress, Ys = yield of the genotypes under limited-irrigation conditions, $\overline{{\mathrm{Y}}} _{{\mathrm{p}}}$  = yield mean under well-watered, and $\overline{{\mathrm{Y}}} _{{\mathrm{s}}}$ = yield mean under limited-irrigation conditions.

### Water productivity (WP)^44^

$${\mathrm{WP}} = \frac{{{\mathrm{Grain}}\:{\mathrm{yield}}\:({\mathrm{kg}}\:{\mathrm{ha}}^{{ - 1}} )}}{{{\mathrm{water}}\:{\mathrm{applied}}\:({\mathrm{m}}^{{\mathrm{3}}} \:{\mathrm{ha}}^{{ - 1}} )\:}}$$

### Statistical analysis

In the M3 generation, genotypes were arranged using a randomized complete block design (RCBD) with three replications. In the M4 and M5 generations, the experiments were produced using a split-plot design with three replications to evaluate the main effects of two factors and their interaction. Irrigation regimes were assigned to the main plots, while genotypes were assigned to the sub-plots.

Mean comparisons were conducted using Fisher’s protected least significant difference (LSD) test at the 0.05 probability level. Tukey test was used to confirm multiple comparisons among genotypes. The phenotypic (PCV%) and genotypic (GCV%) coefficients of variation were estimated according to the method developed by Burton^45^. Broad-sense heritability (hb%) and genetic advance (GA) as a percentage of the trait mean were estimated following the procedures developed by Burton and DeVane^46^. Actual genetic gain (AG %) was estimated as % increase or decrease in yield (GY) of each mutant line relative to the highest yielding mother cultivar.

$$AG\% = \frac{{GY\:of\:mu\tan t\:line - GY\:of\:the\:highest\:yielding\:mother\:cultivar}}{{GY\:of\:the\:highest\:yielding\:mother\:variety}}\: \times \:100$$

A simple correlation was estimated to form a selection index of the studied traits to detect high-yielding and water stress-tolerant wheat lines. Principal component analysis (PCA) was estimated using a correlation matrix to examine relationships between genotypes and the studied traits. GGE biplot analysis estimated to visualize genotype × environmental interactions and identify high-yielding and stable mutant lines across irrigation regimes. Data were analyzed using GENSTAT (18th Edition.VSN International, Hempstead, UK).

## Results

Three generations (M3, M4 and M5) were evaluated under both well-watered and limited-irrigation conditions, except for the M3 generation, which was evaluated only under well-watered conditions.

### M3 generation

#### Phenotypic variation

Data presented in Table 1 revealed significant difference (*P* ≤ 0.05) among wheat genotypes for the studied traits. The mean squares due to genotypes were substantially higher than the corresponding error mean squares for all traits. The coefficient of variation ranged from 10.5% for plant height to 20.0% for spike yield.

**Table 1:** Mean squares from analysis of variance for the studied traits in the M3 generation (2022 season) under well-watered conditions.

Source of variation	PH (cm)	S L (cm)	SW(g)	SY(g)	GS	GWT(g)	SN	GY (t ha^−1^)
Genotypes	900.3**	40.4**	9.8**	7.6**	4625.8**	350.3**	3895.6**	7.8**
Error	55.0	3.21	1.38	1.12	810.4	38.7	520.3	1.37
CV%	10.5	11.7	17.0	19.0	20.	13.9	17.7	19.4

Plant height (PH, cm), Spike length (SL, cm), Spike weight (SW, g),Spike yield (SY, g), grains per spike (GS),1000-grain weight (GWT, g), spikes m^−2^ (SN), Grain yield (GY t ha^−1^). *: significant level at 0.05, CV%: coefficient of variation.

#### Mean performance

The evaluated mutant lines exhibited wide variability for the studied traits in the M3 generation (Table 2). Plant height exhibited the widest range (62.0–108.9 cm), with an average of 82.5 cm. Three mutant lines, namely G5 (62.0 cm), G6 (65.0 cm), and G39 (66 cm), were shorter than their respective mother cultivars.

**Table 2:** Mean performance and range of the studied traits for 40 selected mutant lines and their respective mother cultivars (P1-P5) in the M3 generation (2022 season) under well-watered conditions.

Genotype	PH (cm)	SL (cm)	SW(g)	SY(g)	GS	GWT(g)	SN	GY (t ha^−1^)
Sakha 93 (P1)	96.3	11.8	6.8	5.5	100.0	49.0	125.0	6.10
G1	75.0	16.0	8.1	6.5	130.0	50.0	133.3	8.70
G2	97.2	17.0	12.8	9.5	134.6	60.5	77.9	7.40
G3	96.1	17.0	10.9	7.9	124.7	63.5	91.4	7.20
G4	107.5	13.0	6.0	5.3	120.5	44.0	133.3	7.10
G5	62.0	17.0	11.0	7.6	109.9	60.0	90.7	6.90
G6	65.0	17.0	6.8	5.8	116.0	50.0	117.6	6.80
G7	79.1	18.0	11.0	7.4	112.5	60.0	90.9	6.70
G8	85.0	11.5	5.0	3.8	130.4	33.0	156.5	6.70
G9	88.0	19.0	7.1	5.2	104.6	50.0	126.4	6.60
G10	99.0	19.0	7.7	5.6	123.1	45.5	116.3	6.52
G11	93.0	17.0	5.9	4.8	85.3	56.5	134.7	6.50
G12	84.0	13.0	7.0	5.6	127.3	44.0	114.3	6.45
G13	72.0	14.0	7.0	5.0	142.9	35.0	128.6	6.46
G14	80.0	19.0	6.8	4.8	107.9	44.5	132.7	6.45
G15	79.2	17.0	5.9	4.2	105.5	40.0	151.8	6.44
G16	95.0	17.0	6.5	5.2	95.4	54.5	124.0	6.43
G17	70.5	15.0	6.2	4.7	94.0	49.3	137.3	6.42
G18	76.0	17.0	8.1	6.3	144.8	43.5	99.3	6.36
G19	67.0	17.0	7.0	5.5	110.0	50.0	114.3	6.35
X	82.7	16.3	7.7	5.8	116.8	50.4	119.5	6.76
Sids 13 (P2)	97.3	10.5	7.8	6.6	108.0	47.0	122.0	6.20
G20	68.0	16.0	8.0	6.3	148.2	42.5	100.0	6.34
G21	73.9	12.0	5.2	4.1	102.5	40.0	153.8	6.35
G22	91.8	14.0	3.7	2.9	70.7	41.0	216.2	6.36
G23	76.2	12.0	5.2	4.1	102.5	40.0	153.8	6.37
G24	69.4	14.0	3.7	2.9	70.7	41.0	216.2	6.38
G25	90.0	20.0	5.5	3.9	85.6	45.0	163.6	6.37
X	78.2	14.7	5.2	4.0	96.7	41.6	167.3	6.36
Giza 168 (P3)	90.0	11.5	9.0	7.6	110.0	49.0	112.0	6.30
G26	80.0	20.0	6.0	4.2	71.2	59.0	150.0	6.30
G27	88.0	18.0	7.1	5.0	100.0	50.0	126.8	6.31
G28	108.9	19.0	7.8	5.5	110.0	50.0	115.1	6.33
G29	81.3	15.0	6.2	4.8	112.9	42.5	129.0	6.28
G30	96.0	15.0	5.6	4.2	107.7	39.0	144.1	6.20
G31	82.0	14.0	6.6	5.0	102.0	49.0	122.0	6.20
G32	80.0	14.0	6.2	4.7	120.5	39.0	129.0	6.13
X	88.0	16.4	6.5	4.8	103.5	46.9	130.9	6.30
Gemmeiza-9 (P4)	86.5	12.5	8.0	6.9	108.0	52.0	114.0	6.40
G33	75.0	14.0	5.0	3.8	102.7	37.0	160.0	6.20
G34	77.0	13.0	5.6	4.3	86.0	50.0	142.9	6.22
G35	85.0	19.0	5.6	3.8	76.0	50.0	160.7	6.12
G36	75.0	14.0	8.0	6.0	134.8	44.5	100.0	6.11
X	78.0	15.0	6.1	4.5	99.9	45.4	140.9	6.16
Maryout 5 (P5)	105.0	13.5	7.4	5.7	91.1	51.0	121.0	5.60
G37	80.0	16.0	8.0	6.0	120.0	50.0	100.0	6.00
G38	95.0	19.0	6.1	4.6	97.9	47.0	131.1	6.12
G39	66.0	13.0	5.5	4.0	90.9	44.0	146.8	6.00
G40	80.0	16.0	8.9	6.5	129.2	50.0	67.6	6.00
X	80.3	16.0	7.1	5.3	109.5	47.8	111.4	6.03
Range	62.0-108.9	11.5–20.0	3.7–12.8	2.9–9.5	70.7-148.2	33.0-70.5	67.6-216.2	5.9–8.7
LSD _0.05_	12.4	2.6	2.3	1.8	53.0	9.9	55.0	0.25

Plant height (PH, cm), Spike length (SL, cm), Spike weight (SW, g),Spike yield (SY, g), grains per spike (GS),1000-grain weight (GWT, g), spikes m^−2^ (SN), Grain yield (GY t ha^−1^), LSD _0.05_:least significant difference.

**Fig. 2:**
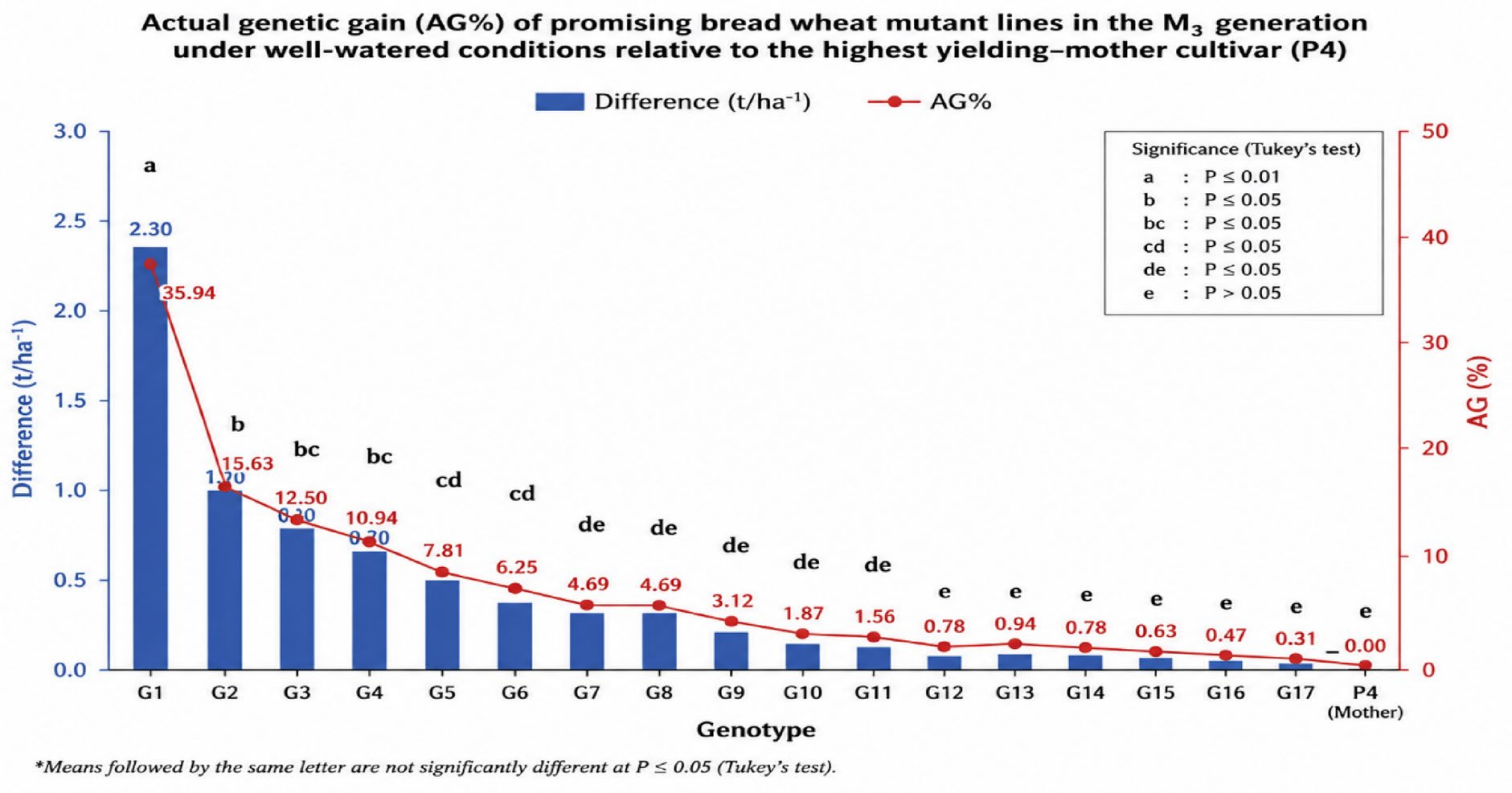
Actual genetic gain (AG%) of mutant lines in the M3 generation (2022) under well-watered conditions relative to the best cultivar (P4).

For spike length, thirty mutant lines exceeded the mean values of their respective mother cultivars. Mutant lines, G25 and G26 recorded the longest spikes (20.0 cm), followed by mutant lines G9, G10, G14, G28, G35, and G38 (19.0 cm). Regarding spike weight, considerable variation was recorded, ranged from 3.7 to 12.8 g, with G2 recorded the highest value. Mutant lines G2, G5, and G7 significantly outperformed their respective mother cultivars for this trait. For spike yield, mutant line G2 achieved the highest value (9.5 G), representing a 46.2% enhancement over its mother cultivar. In addition, four mutant lines (G3, G5, G7, and G18) showed superior performance compared with their mother cultivars. Regarding grains per spike, two mutant lines (G20:148.2 and G18:144.8) exhibited the highest values relative to their mother cultivars. With respect to 1000-grain weight, mutant line G2 produced the heaviest (60.5 g), followed by G5 and G7 (60.0 g). Mutant lines G7 and G24 achieved the highest spikes m^−2^. The top-performing genotypes for grain yield included G1, G2, G3, and G4. Figure 2 illustrated significant variation according to Tukey’s test in actual genetic gain (AG %) among the promising wheat mutant lines in the M3 generation under well-watered conditions. A gradual decline in AG% was observed across the evaluated wheat mutant lines. Several mutant lines (G1-G11) significantly outperformed the highest-yielding mother cultivar (P4) in grain yield, whereas mutant lines G12-G17 did not differ significantly from P4. The observed enhancements in grain yield among the mutant lines ranged from 35.94% in G1 to 0.31% in G17.

### M4 generation

#### Genotype × environment interaction

The analysis of variance in the M4 revealed highly significant effects (*P* < 0.05) of genotypes (G), irrigation regimes (T), and their interaction (G × T) on all studied traits under both well-watered and limited-irrigation conditions (Table 3).

**Table 3:** Mean squares from analysis of variance (split-plot design) showed the effects of irrigation regimes, genotypes, and their interaction on all studied traits in the M4 generations (2023 season) under well-watered and limited-irrigation conditions.

Source of variation	PH(cm)	SL(cm)	SW(g)	SY(g)	GS	GWT (g)	SN	GY (t ha^−1^)
Treatments	624.2**	64.5**	123.4**	411.1**	258703.3**	170079.7**	91262.6**	345.2**
Error **(**a**)** Rep×T	1.0	0.08	1.10	49.6	200.7	44.2	919.6	0.001
Genotypes	119.5**	2.5^ns^	4.4**	106.0**	298.0**	387.3**	2108.2**	5.3**
T × G	146.8**	3.2**	3.9**	125.0**	306.3**	380.7**	1430.1**	2.9**
Error (b)	24.1	1.7	1.4	39.2	146.7	99.6	173.6	0.7

*Significant levels at 0.05, ns: non-*significant.

#### Mean performance

Table 4 showed the mean performance and range of the studied traits for the 20 selected wheat mutant lines in the M_4_ generation, along with their mother cultivars, under well-watered and limited-irrigation conditions. Across this generation, all studied traits were substantially reduced under limited-irrigation conditions.

**Table 4:** Mean performance and range of the studied traits for the selected mutant lines and their mother cultivars in the M4 generation (2023 season) under well-watered (Yp) and limited-irrigation conditions (Ys).

Genotype	Condition	PH (cm)	S L (cm)	SW(g)	SY(g)	GS	GWT(g)	SN	GY (t ha^−1^)
Sakha 93 (P1)	Yp	105.0	11.0	6.5	5.2	98.0	51.0	122.0	6.0
	Ys	88.0	11.8	4.9	3.5	85.0	49.4	75.0	3.1
	$\overline{X}$	96.5	11.4	5.7	4.4	91.5	50.2	98.5	4.6
G1	Yp	98.0	17.0	9.1	7.8	121.0	57.0	134.0	9.2
	Ys	68.0	14.0	6.1	4.8	96.0	43.0	119.0	4.9
G2	Yp	105.0	14.0	7.4	6.4	123.5	60.0	121.0	9.0
	Ys	94.5	11.0	4.4	3.4	98.5	47.0	106.0	4.9
G3	Yp	94.0	16.0	8.5	6.8	104.0	56.0	152.0	8.9
	Ys	70.0	13.0	5.5	3.8	79.0	40.5	137.0	4.4
G4	Yp	97.0	15.0	8.0	6.5	100.0	55.0	159.0	8.8
	Ys	80.0	12.0	5.0	3.5	73.0	40.0	144.0	4.2
G5	Yp	78.5	14.0	7.4	6.4	123.3	59.0	120.0	8.7
	Ys	65.3	11.0	4.4	3.4	99.0	41.0	105.0	4.3
G6	Yp	95.0	16.0	8.5	7.2	108.0	58.0	138.0	8.6
	Ys	85.0	13.0	5.5	4.2	80.0	43.0	115.0	4.0
G7	Yp	118.0	15.0	8.0	6.6	110.0	53.0	145.0	8.5
	Ys	82.8	12.0	5.0	3.6	83.0	42.0	111.0	3.9
G8	Yp	100.0	15.0	8.0	6.8	132.3	57.0	111.2	8.4
	Ys	74.8	12.0	5.0	3.8	106.0	43.0	84.0	3.8
G9	Yp	110.0	15.0	8.0	6.5	105.0	56.0	141.0	8.3
	Ys	85.0	12.0	5.0	3.5	79.0	43.0	107.0	3.6
G10	Yp	115.0	14.0	7.4	6.4	101.0	60.0	133.0	8.1
	Ys	83.6	11.0	4.4	3.4	71.0	43.0	117.0	3.6
Sids 13 (P2)	Yp	108.0	12.0	7.0	5.4	101.3	49.0	124.0	6.2
	Ys	81.0	12.6	5.8	4.7	82.0	48.4	78.0	3.0
	$\overline{X}$	94.5	12.3	6.4	5.1	91.7	48.7	101.0	4.6
G11	Yp	105.0	15.0	8.0	6.9	108.0	59.0	125.0	8.0
	Ys	77.0	12.0	5.0	3.9	85.0	45.0	92.0	3.5
G12	Yp	100.0	15.0	8.0	6.8	123.0	58.0	106.8	7.6
	Ys	85.0	12.0	5.0	3.8	75.0	40.0	112.0	3.4
G13	Yp	95.2	16.0	8.5	7.3	103.0	58.0	124.0	7.4
	Ys	77.0	13.0	5.5	4.3	85.0	40.0	89.0	3.0
Giza 168 (P3)	Yp	111.0	11.8	7.6	6.1	108.0	49.0	122.0	6.5
	Ys	84.0	11.6	5.6	3.3	77.0	51.0	81.0	3.2
	$\overline{X}$	97.5	11.7	6.6	4.7	92.5	50.0	101.5	4.9
G14	Yp	112.0	17.3	9.3	7.7	111.0	60.5	110.0	7.3
	Ys	83.9	14.1	6.3	4.7	86.0	45.0	80.0	3.1
G15	Yp	104.0	16.0	8.5	7.3	113.0	59.0	108.0	7.2
	Ys	75.2	10.0	3.9	2.7	76.0	41.0	100.0	3.1
G16	Yp	105.0	12.0	6.4	5.5	105.8	57.0	118.0	7.1
Gemmeiza-9 (P4)	Yp	116.0	12.3	6.9	5.3	100.0	50.0	130.0	6.5
	Ys	81.0	11.6	5.6	4.3	70.0	51.0	84.0	2.9
	$\overline{X}$	98.5	12.0	6.3	4.8	85.0	50.5	107.0	4.7
	Ys	72.4	10.0	3.9	2.6	82.0	42.0	90.0	3.1
G17	Yp	117.0	16.0	8.5	7.4	102.0	59.0	116.0	7.0
	Ys	79.2	10.0	3.9	2.8	82.0	40.0	95.0	3.1
G18	Yp	100.0	13.0	6.9	5.8	107.0	55.0	113.0	6.7
	Ys	82.0	13.0	5.5	4.3	95.0	42.0	75.0	3.0
Maryout 5 (P5)	Yp	114.0	13.2	7.3	5.7	103.0	52.0	119.0	6.4
	Ys	87.0	10.8	6.2	4.9	78.0	50.5	74.0	2.9
	$\overline{X}$	100.5	12.0	6.8	5.3	90.5	51.3	96.5	4.7
G19	Yp	100.0	13.0	6.9	5.7	101.0	60.0	108.0	6.5
	Ys	87.1	9.0	3.4	2.5	80.8	37.5	93.0	2.8
G20	Yp	98.0	13.0	6.9	5.6	107.0	54.0	112.0	6.5
	Ys	88.0	13.0	5.5	4.4	77.0	39.5	95.0	2.9
Grand mean	Yp	102.3	14.9	7.9	6.7	110.5	57.5	124.8	7.9
	Ys	81.1	11.9	4.9	3.7	84.4	41.9	103.3	3.63
Range	Yp	94.0-118.0	12.0–17.0	6.4–9.1	5.5–7.8	100.0-132.0	53.0–60.0	106.8–159.0	6.5–9.2
	Ys	65.3–94.5	9.0-14.1	3.4–6.3	2.5–4.8	71.0-106.0	37.5–47.0	75.0-144.0	2.8–4.91
CV %	Yp	9.8	11.9	11.6	11.1	7	14.8	4.1	19.3
	Ys	8.0	10.0	9.0	11.0	15.0	19.0	9.0	18.0
LSD _0.05_	Yp	7.20	1.60	2.10	1.60	9.00	6.00	4.20	0.20
LSD _0.05_	Ys	2.70	1.00	0.90	0.70	3.10	3.00	12.00	0.60

Plant height (PH, cm), Spike length (SL, cm), Spike weight (SW, g),Spike yield (SY, g),grains per spike (GS),1000-grain weight (GWT, g),Spikes m^−2^ (SN), Grain yield (GY t/ha^−1^),CV%: coefficient of variation, mother cultivars (P1 to P5).LSD _0.05_: least significant different.

**Fig. 3:**
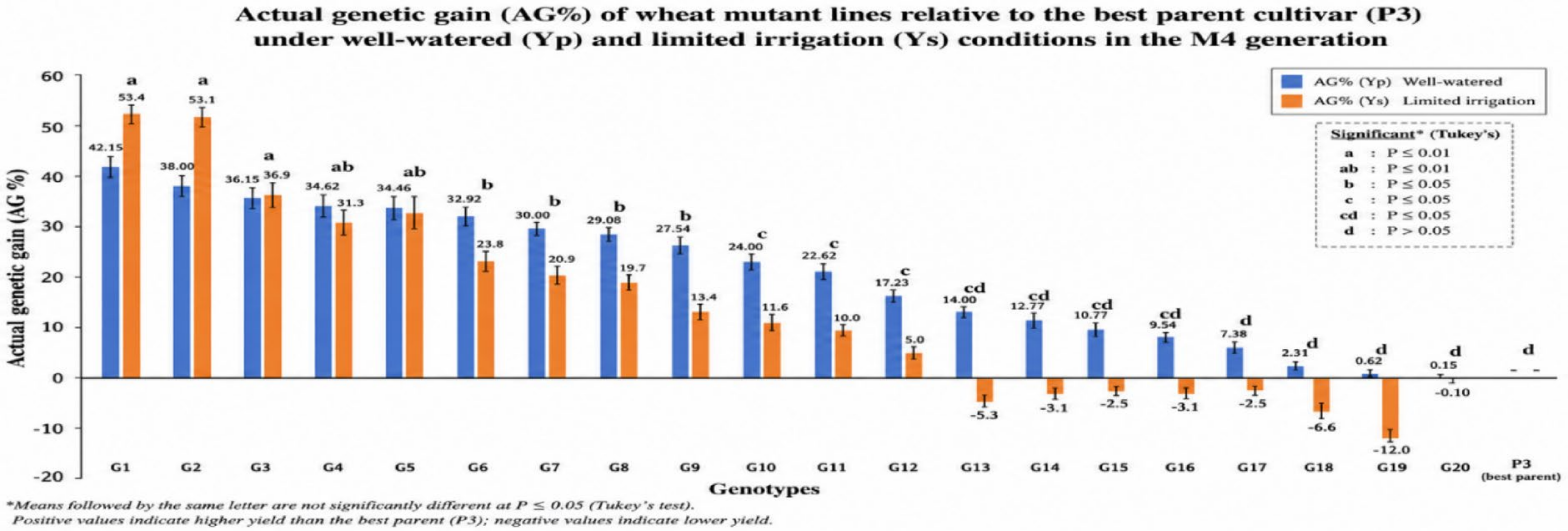
Actual genetic gain (AG%) of mutant lines in the M4 generation relative to the mother cultivars under well-watered (Yp) and limited-irrigation (Ys) conditions.

Under well-watered conditions, all mutant lines outperformed their respective mother cultivars, particularly for grain yield and its contributing components. Several mutant lines (G1, G2, G3, G4, G6, and G14) exhibited superior performance compared with their mother cultivars. Mutant line G5 was shorter than its respective mother cultivar. Mutant line G14 recorded the highest spike length, heavier spike weight, spike yield, and 1000-grain weight. Additionally, mutant line G2 exhibited the highest grains per spike and 1000-grain weight. Under limited-irrigation conditions, substantial reductions were recorded in all yield-related traits. Grain yield showed a mean reduction of 51.3% compared with well-watered conditions. Nevertheless, several mutant lines maintained relatively higher values than their mother cultivars. The wide phenotypic ranges observed among mutant lines. Figure 3 showed actual genetic gain (AG %) for grain yield of mutant lines relative to the highest-yielding mother cultivar (P3) under both conditions in the M4 generation. Under well-watered conditions, AG% ranged from 42.15% in G1 to 0.15% in G20. Three mutant lines (G1-G3) outperformed the highest-yielding mother cultivar (P3) and formed the top statistical group (a). Under limited-irrigation conditions, the same mutant lines maintained high AG % and stable genetics, remaining within the top statistical group (a).

#### GGE biplot analysis

**Fig. 4:**
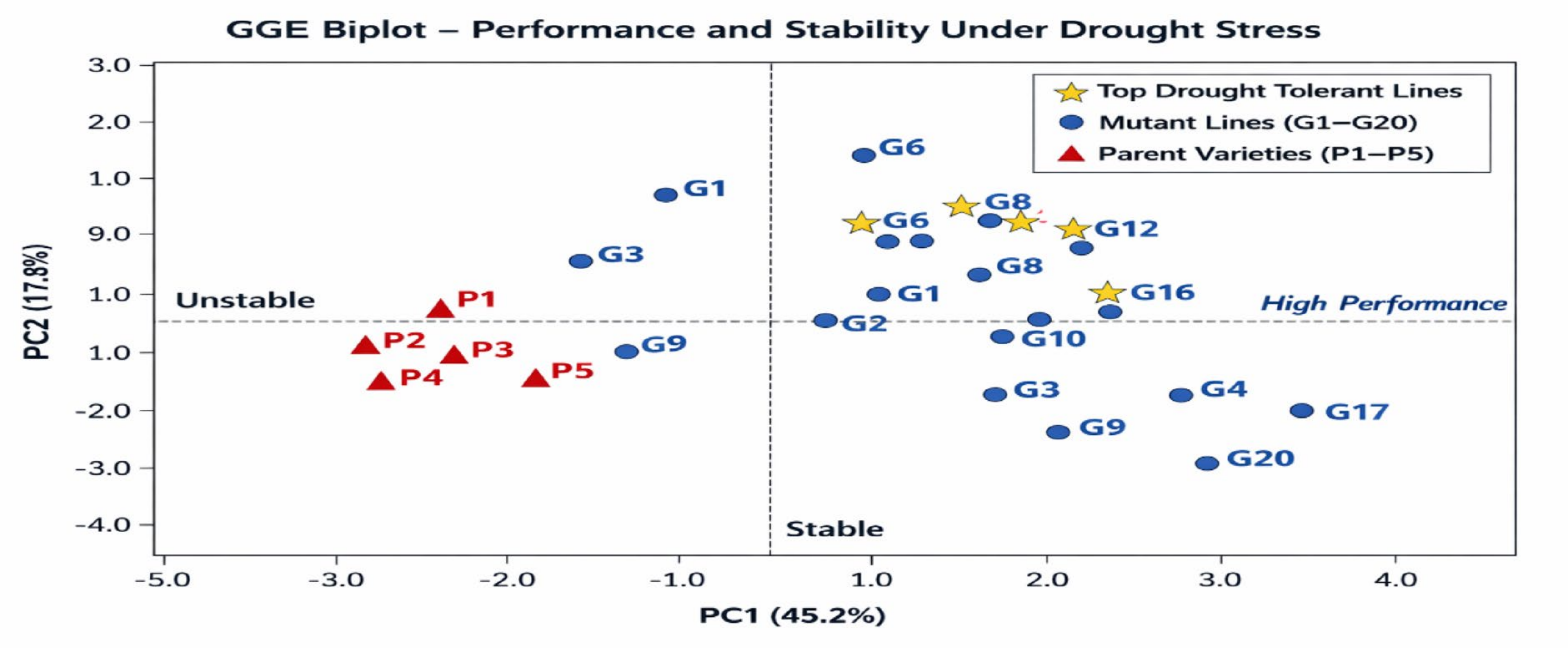
GGE biplot illustrated productivity performance (PC1) and stability (PC2) of mutant lines in the M4 generation, along their mother cultivars.

The GGE Biplot analysis revealed that the first two principal components (PC1 and PC2) interpreted 77.3% of the total variation (Fig. 4).

The biplot separated the wheat genotypes into distinct groups based on their performance and stability under limited-irrigation conditions. PC1 (the right side of the plot) primarily represented mean productivity; whereas PC2 represented genotype × environment interaction (reflected stability). The PCA scores separated the mutant lines from their mother cultivars under both conditions based on the studied traits. Mutant lines G6, G8, G12, and G16 recorded the highest PC1 scores. Mutant lines G3, G4, G19, and G20 were located on the negative side of PC1. The mother cultivars recorded negative PC2 values and relatively low PC1 scores, except P2 and P4.

###  M5 generation

#### Genotype-environment interaction

The analysis of variance for the M5 generation revealed significant effects (*P* ≤ 0.05) of genotypes (G), irrigation regimes (T), and G × T interaction on all studied traits under well-watered and limited-irrigation conditions (Table 5).

**Table 5:** The mean squares from the analysis of variance (split-plot design) showed the effects of genotypes, irrigation regimes, and their interaction on all studied traits in the M5 generation (2024 season) under well-watered and limited-irrigation conditions.

Source of variation	PH(cm)	SL(cm)	SW(g)	SY(g)	GS	GWT (g)	SN	GY (t ha^−1^)
Treatments	3715.6**	49.6**	19.3**	1.8**	1982.4**	452.6**	41576.8**	315.6**
Error (a) Rep×T	17.8	1.8	0.49	0.10	10.3	2.6	50.1	2.6
Genotypes	1489.5**	9.5**	6.7**	2.12**	1274.5**	284.5**	10864.3**	214.1**
T × G	152.1**	1.4**	1.02**	0.25**	157.8**	39.1**	1152.5**	31.5
Error (b)	28.2	0.47	0.31	0.07	101.2	39.8	471.8	6.2

*Significant levels at 0.05, Plant height (PH, cm), Spike length (SL, cm), Spike weight (SW, g),Spike yield (SY, g),grains per spike (GS),1000-grain weight (GWT, g),Spikes m^−2^ (SN), Grain yield (GY t ha^−1^).

#### Mean performance

In this generation, twelve selected mutant lines in the M5 generation were evaluated under both conditions. The results presented in Table 6 revealed considerable variations among wheat genotypes.

**Table 6:** Mean performance and range of the studied traits for twelve selected mutant lines in the M5 generation (2024 season) and their mother cultivars under well-watered (Yp) and limited-irrigation conditions (Ys).

Genotype	Condition	PH (cm)	SL(cm)	SW(g)	SY(g)	GS	GWT (g)	SN	GY(t ha^−1^)
Sakha 93 (P1)	Yp	90.0	10.8	7.0	5.90	112.0	49.0	115.0	6.3
	Ys	95.0	11.0	4.5	3.20	80.0	48.0	108.0	4.2
	X	92.5	10.9	5.8	4.60	96.0	48.5	111.5	5.3
G1	Yp	99.30	13.70	7.30	5.80	102.10	56.30	140.00	8.00
	Ys	70.00	13.10	7.50	5.60	109.10	49.90	106.70	5.10
G2	Yp	84.60	9.00	4.30	3.40	72.00	52.80	200.00	7.60
	Ys	77.00	11.90	6.30	4.60	84.00	53.80	112.20	5.00
G3	Yp	112.00	15.70	11.49	7.40	133.30	40.00	140.00	7.50
	Ys	79.00	12.20	6.90	4.90	97.70	51.90	106.80	4.90
G4	Yp	89.10	12.50	11.17	5.70	95.70	53.50	126.90	6.50
	Ys	85.00	12.30	7.10	5.10	97.70	53.00	103.70	5.00
G5	Yp	72.50	10.40	14.59	9.20	85.30	53.60	148.30	6.80
	Ys	70.20	12.10	8.20	5.90	93.40	55.60	108.60	4.90
Sids 13 (P2)	Yp	88.2	12.6	8.20	6.8	113.0	44.0	117.0	5.80
	Ys	85.0	12.0	5.8	4.3	85.0	47.0	110.0	4.30
	X	86.6	12.3	7.0	5.6	99.0	45.5	113.5	5.10
G6	Yp	80.80	11.10	6.20	4.60	80.20	57.40	150.30	6.90
	Ys	77.00	12.50	7.60	5.30	95.80	56.60	81.80	4.30
G7	Yp	81.90	12.00	6.10	4.80	74.00	59.80	174.60	7.70
	Ys	80.10	12.30	7.00	4.90	95.50	54.10	80.50	4.20
G8	Yp	79.40	12.40	6.7	4.70	78.90	58.10	156.90	7.20
	Ys	75.40	10.80	5.40	3.8	65.10	51.30	121.50	4.60
Giza 168 (P3)	Yp	95.4	11.0	5.9	5.0	115.0	51.0	105.3	6.20
	Ys	90.0	9.0	5.1	3.0	90.0	58.0	95.0	4.90
	X	92.7	10.0	5.5	4.0	102.5	54.5	100.2	5.60
G9	Yp	78.00	10.50	5.90	4.20	77.90	55.80	150.40	6.50
	Ys	75.00	11.50	6.30	4.50	91.20	55.20	82.40	4.10
G10	Yp	77.50	10.80	12.00	8.90	89.10	51.10	178.00	8.10
	Ys	74.00	9.0	6.90	4.90	81.40	50.10	132.90	4.70
Gemmeiza-9 (P4)	Yp	82.8	13.0	7.7	6.3	113.0	50.0	108.0	6.10
	Ys	78.0	11.7	6.0	4.6	80.0	53.0	95.0	4.0
	X	80.4	12.4	6.9	5.5	96.5	51.5	101.5	5.10
	Ys	74.00	10.90	6.90	4.90	81.40	50.10	132.90	5.40
G11	Yp	84.40	10.0	6.5	5.30	79.80	50.50	198.50	8.00
	Ys	68.20	9.2	4.7	3.90	63.20	51.10	126.30	4.0
Maryout 5 (P5)	Yp	94.5	10.8	7.0	5.9	108.0	47.0	108.0	5.8
	Ys	94.0	11.0	5.7	4.5	75.0	49.0	95.0	3.5
	X	94.3	10.9	6.4	5.2	91.5	48.0	101.5	4.7
G12	Yp	76.10	9.60	5.00	4.10	77.60	54.90	197.20	8.40
	Ys	74.90	10.30	6.10	4.20	74.70	53.50	123.60	4.90
X	Yp	82.80	11.40	7.92	4.80	87.20	53.70	163.40	7.40
	Ys	81.60	11.60	5.40	3.90	85.00	51.00	104.60	4.50
Range	Yp	64.4–112.0	9.0-15.7	4.3–14.5	3.4–9.2	72.0-133.3	40.0-59.8	126.9–200.0	6.5–8.1
	Ys	70.0–85.0	10.3–13.1	5.7–8.2	3.9–5.9	73.2-109.1	49.9–56.6	80.5-123.9	4.1–5.4
CV%	Yp	4.0	7.0	15.5	16.0	14.4	14.0	17.0	12.0
	Ys	6.0	10.0	13.5	18.0	16.4	14.0	13.0	10.0
Mean	Yp	100.2	12.6	8.0	6.6	108.9	49.0	115.8	6.2
	Ys	89.1	10.4	6.6	5.4	70.8	46.7	92.8	3.1
LSD _0.05_	Yp	13.2	1.7	1.0	0.9	12.1	8.2	35.9	1.0
LSD _0.05_	Ys	6.7	0.85	0.3	0.23	8.1	4.8	5.3	0.32

Plant height (PH, cm), Spike length (SL, cm), Spike weight (SW, g),Spike yield (SY, g), grains per spike (GS),1000-grain weight (GWT, g), spikes m^−2^ (SN), Grain yield (GY t/ha^−1^),SL: Significant level at 0.05,CV%: coefficient of variation, mother cultivars (P1 to P5).LSD _0.05_: least significant different.

**Fig. 5:**
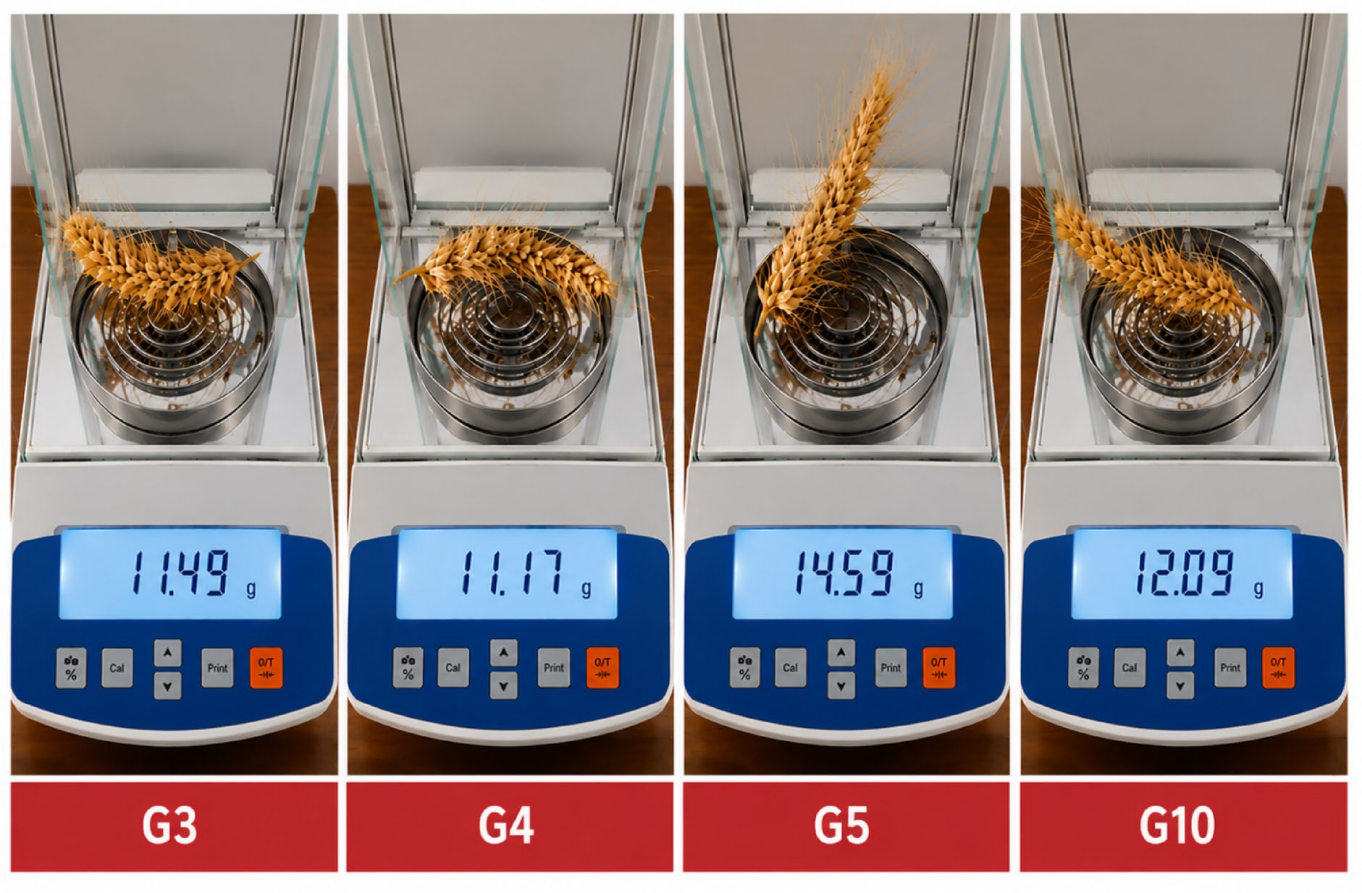
Spike weights of selected M5 wheat mutant lines ( G3, G4, G5, and G10) under well-watered conditions.

The mutant lines outperformed their mother cultivars for all traits except plant height. In particular, mutant lines G3, G4, G5, and G10 exhibited higher spike weight compared with their mother cultivars (Fig. 5).

**Fig. 6:**
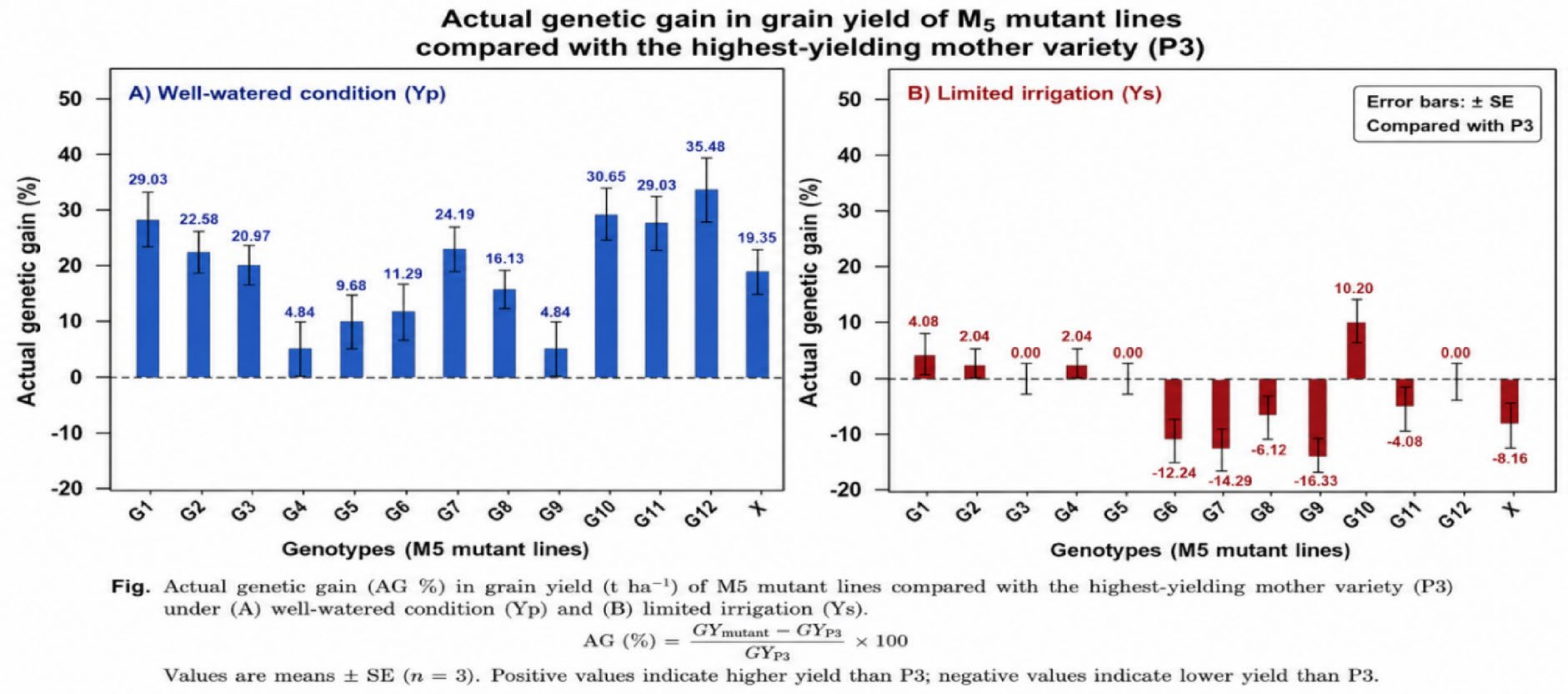
Actual genetic gain (AG%) in grain yield of mutant lines in the M5 generation relative to the highest-yielding mother cultivar (P3) under both conditions.

Mutant line G12 showed the highest AG% (35.48%) relative to the highest-yielding mother cultivar (P3), followed by G10 (30.65%), G11 (29.03%), and G1 (29.03%). In contrast, under Ys, mutant line G10 showed the highest genetic gain (10.20%), followed by G1 (4.08%), G2 (2.04%), and G4 (2.04%) (Fig. 6).

The coefficient of variation (CV%) values were low (10) to moderate (10–20%) for most traits. Generally, all traits decreased under limited-irrigation conditions. Mutant lines showed relatively higher grain yield under limited-irrigation conditions compared with their mother cultivars. The CV% values were generally moderate (10–20%). Three mutant lines, called G2, G10, and G12, exhibited superior grain yield compared to the best mother (P3) under both conditions.

#### Principal components and GGE biplot

**Fig. 7:**
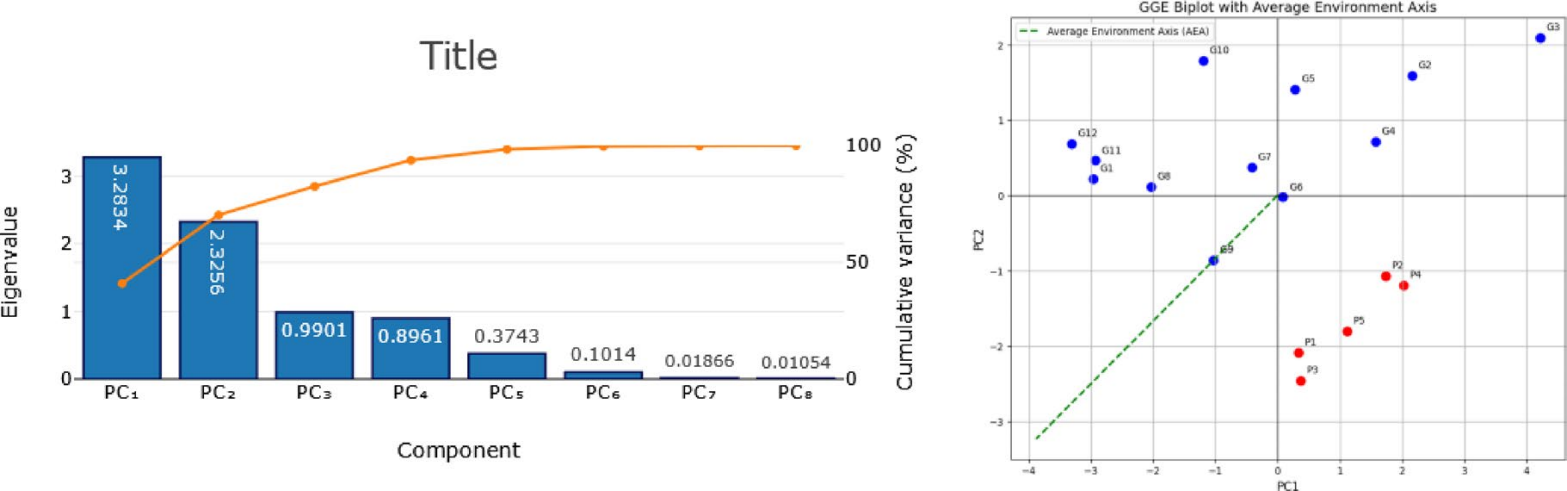
Principal components analysis (PCA) and GGE biplot based on the studied traits of wheat mutant lines in the M5 generation with their mother cultivars.

Figure 7 showed two complementary analyses: eigenvalue/PCA (left side) and GEE biplot analysis (right side). These analyses described the pattern of variation and genotype performance under both conditions. The first two principal components (PC1 and PC2) explained 77.3% of the total variance.

The GGE biplot separated wheat genotypes based on yield mean and stability. Mutant lines G2, G3, G4, G5, and G6 were located on the positive side of PC1 and exhibited moderately positive PC2 values. Mutant lines G4, G7, G10, G11, and G12 were closer to the origin. In contrast, the mother cultivars were positioned in the lower half of the biplot and had positive PC1 scores. Three mutant lines, G6, G9, and G7, were located nearest to the origin point (more stable).

### Genetic parameters

The results presented in Table 7 demonstrated substantial variation in genetic parameters among the studied traits across the M3 and M4 generations.

**Table 7:** Genetic parameters of the studied traits across the M3 and M4 generations of wheat mutant lines.

	M3 generation				M4 generation							
	Well-watered conditions				Well-watered conditions				Limited-irrigation conditions			
Trait	PCV	GCV	hb	GA	PCV	GCV	hb	GA	PCV	GCV	hb	GA
PH (cm)	21.0	20.3	93.9	40.6	17.6	14.8	78.0	18.0	14.3	11.6	72.0	20.0
SL (cm)	23.1	22.1	92.1	43.8	18.7	16.3	72.0	16.0	16.7	13.8	75.0	24.3
SW (g)	26.2	24.3	85.9	46.4	24.8	22.5	81.0	22.0	22.4	18.9	73.0	32.5
SY (g)	30.6	28.3	85.3	53.8	26.8	24.1	79.0	24.0	25.6	21.7	68.0	30.7
GS	36.0	32.7	82.5	61.2	32.1	28.7	85.0	28.0	20.1	17.4	74.0	31.1
GWT(g)	22.7	21.4	89.0	41.5	18.4	15.2	74.0	17.0	19.5	16.2	77.0	29.3
SN	27.7	25.8	86.6	49.5	33.2	30.5	88.0	30.0	22.6	19.8	78.0	36.4
GY (t ha^−1^)	25.2	22.9	82.4	42.8	28.8	26.4	82.0	26.0	26.1	23.4	76.0	41.0

PCV %: phenotypic coefficient of variation, GCV%: genotypic coefficient of variation and hb%:Heritability in the broad sense, GA: genetic advanced from selection as percentage of trait mean. Plant height (PH, cm), Spike length (SL, cm), Spike weight (SW, g),Spike yield (SY, g),grains per spike (GS),1000-grain weight (GWT, g),Spikes m^−2^ (SN), Grain yield (GY t ha^−1^).

In the M4 generation, both phenotypic (PCV) and genotypic (GCV) coefficients of variation values were higher (≥ 20%) than those observed in the M3 generation for most studied traits under well-watered conditions. Heritability estimates were high (≥ 60%) for all studied traits and were coupled with high genetic advance (GA) (≥ 20%). In the M4 generation, the PCV and GCV values were higher for spike weight, spike yield, grains per spike, spikes m^−2^, and grain yield under both conditions. Heritability values remained high (≥ 60%) across most traits and were coupled with moderate to high genetic advance values under both conditions. In the M4 generation under limited-irrigation conditions, some traits reduced variability and heritability estimates compared with well-watered conditions. Nevertheless, GA% remained moderate (10–20%) for grain yield, spikes m^−2^, and spike weight.

### Correlation coefficients

Correlation coefficients revealed strong associations between grain yield and the studied traits under both conditions across generations. Grain yield exhibited positive and significant correlations with spikes m^−2^. Spike length was positively and significantly correlated with both spike weight and spike yield. Spike weight was positively and significantly correlated with spike yield and grains per spike. Under well-watered conditions, the 1000-grain weight was positively and significantly correlated with both spikes m^−2^ and grain yield. Furthermore, spikes m^−2^ were positively and significantly correlated with 1000-grain weight across generations under both conditions, except under limited-irrigation conditions in the M5 generation (Table 8). In general, spikes m^−2^, spike weight, and spike yield were key yield-contributing traits for improving grain yield under both conditions.

**Table 8:** Phenotypic correlation between the studied traits in the M4 and M5 generations under well-watered and limited-irrigation conditions.

	S L (cm)	SW(g)	SY(g)	GS	GWT(g)	SN	GY (t ha^−1^)
M4 generation under well-watered conditions							
PH (cm)	0.014	0.017	0.073	− 0.103	0.172	− 0.063	− 0.094
SL (cm)		0.999*	0.968*	0.122	0.181	0.242	0.392*
SW (g)			0.965*	0.124	0.172	0.238	0.384*
SY (g)				0.208	0.315	0.112	0.365
GS					0.127	− 0.353	0.391*
GWT(g)						0.999**	0.675*
SN							0.676*
M4 generation under limited-irrigation conditions							
PH (cm)	− 0.241	− 0.247	− 0.116	0.261	0.012	− 0.074	0.116
SL (cm)		0.998*	0.969*	0.209	0.362	0.108	0.317
SW (g)			0.967*	0.203	0.363	0.088	0.294
SY (g)				0.315	0.401*	− 0.011	0.291
GS					0.388*	− 0.443*	0.347
GWT(g)						0.388*	0.443*
SN							0.639*
M5 generation under well-watered conditions							
PH (cm)	0.860*	0.619*	0.770*	0.862*	− 0.661*	− 0.37	0.301
SL (cm)		0.775**	0.891**	0.885*	− 0.519*	− 0.627*	0.202
SW (g)			0.957**	0.899*	− 0.744*	− 0.647*	− 0.025
SY (g)				0.932*	− 0.682*	− 0.642*	0.108
GS					− 0.823*	0.561*	0.174
GWT(g)						0.979*	0.502*
SN							0.504*
M5 generation under limited-irrigation conditions							
PH (cm)	− 0.057	− 0.379	− 0.470	− 0.076	0.069	− 0.212	− 0.113
SL (cm)		0.533*	0.615*	0.924*	− 0.064	− 0.293	0.323
SW (g)			0.970*	0.486*	0.180	− 0.007	0.241
SY (g)				0.570*	0.050	0.092	0.400
GS					− 0.140	− 0.353	0.229
GWT(g)						− 0.140	0.353
SN							0.775*

Plant height (PH, cm), Spike length (SL, cm), Spike weight (SW, g),Spike yield (SY, g), grains per spike (GS),1000-grain weight (GWT, g), spikes m^−2^ (SN), Grain yield (GY t ha^−1^), *: significant level at 0.05.

### Drought indices

Table 9 illustrated wide variation among the selected mutant lines for drought indices across the M4 and M5 generations under both conditions. In the M4 generation, mutant lines G1-G8 exhibited higher values of mean productivity (MP), geometric mean productivity (GMP), harmonic mean (HM), stress tolerance index (STI), and yield index (YI) compared with their mother cultivars under both conditions. Mutant lines G1 and G2 showed higher values of yield stability index (YSI) compared to other genotypes.

**Table 9:** Drought indices of the selected wheat mutant lines in the M4 and M5 generations under well-watered and limited-irrigation conditions.

Genotype		MP	GMP	HM	TOL	STI	SSI	YI	YSI
M4 generation									
G1		7.05	6.71	14.7	4.3	0.72	0.87	1.35	0.533
G2		6.95	6.64	14.7	4.1	0.71	0.84	1.35	0.544
G3		6.65	6.26	13.2	4.5	0.63	0.94	1.21	0.494
G4		6.5	6.08	12.6	4.6	1.29	0.97	1.16	0.477
G5		6.5	6.12	12.9	4.4	0.60	0.94	1.18	0.494
G6		6.3	5.87	12.0	4.6	0.55	0.99	1.10	0.465
G7		6.2	5.76	11.7	4.6	0.53	1.00	1.07	0.459
G8		6.1	5.65	11.4	4.6	1.33	1.01	1.05	0.452
G9		5.95	5.47	10.8	4.7	1.24	1.05	0.99	0.434
G10		5.85	5.40	10.8	4.5	1.51	1.03	0.99	0.444
G11		5.75	5.29	10.5	4.5	1.59	1.04	0.96	0.438
G12		5.5	5.08	10.2	4.2	1.40	1.02	0.94	0.447
G13		5.2	4.71	9.0	4.4	1.39	1.10	0.83	0.405
G14		5.2	4.76	9.3	4.2	1.49	1.07	0.85	0.425
G15		5.15	4.72	9.3	4.1	1.55	1.05	0.85	0.431
G16		5.1	4.69	9.3	4.0	1.70	1.04	0.85	0.437
G17		5.05	4.66	9.3	3.9	1.67	1.03	0.85	0.443
G18		4.85	4.48	9.0	3.7	1.64	1.02	0.83	0.448
G19		4.65	4.27	8.4	3.7	1.57	1.05	0.77	0.431
G20		4.7	4.34	8.7	3.6	2.09	1.03	0.80	0.446
Mother cultivars									
Sakha 93 (P1)		4.5	4.32	4.0	2.9	0.467	0.482	1.016	0.518
Sids 13 (P2)		4.7	4.45	4.2	3.0	0.497	0.484	1.045	0.516
Giza 168 (P3)		4.8	4.56	4.3	3.3	0.521	0.508	1.045	0.492
Gemmeiza-9 (P4)		4.7	4.34	4.0	3.6	0.472	0.554	0.947	0.446
Maryout 5 (P5)		4.6	4.38	3.9	3.5	0.465	0.547	0.947	0.453
M5 generation									
G1		6.55	6.39	15.3	2.9	0.75	0.99	1.09	0.638
G2		6.3	6.16	15.0	2.6	0.70	0.93	1.08	0.658
G3		6.2	6.06	14.7	2.6	0.67	0.94	1.05	0.653
G4		5.75	5.70	15.0	1.5	0.94	0.63	1.07	0.769
G5		5.85	5.77	14.7	1.9	0.61	0.76	1.05	0.721
G6		5.35	5.25	12.9	2.1	0.50	0.89	0.92	0.672
G7		5.95	5.69	12.6	3.5	0.59	1.24	0.90	0.545
G8		5.9	5.75	13.8	2.6	1.38	0.98	0.98	0.639
G9		5.3	5.16	12.3	2.4	1.11	1.00	0.88	0.631
G10		6.6	6.43	15.3	3.0	2.13	1.01	1.09	0.630
G11		6	5.66	12.0	4.0	1.81	1.36	0.86	0.500
G12		6.65	6.42	14.7	3.5	2.23	1.13	1.05	0.583
Mother cultivars									
Sakha 93 (P1)	5.25		5.144	5.04	2.1	0.725	0.333	0.933	0.667
Sids 13 (P2)	5.05		4.994	4.94	1.5	0.684	0.259	0.956	0.741
Giza 168 (P3)	5.55		5.512	5.47	1.3	0.833	0.210	1.089	0.790
Gemmeiza-9 (P4)	5.2		5.122	5.04	1.8	0.719	0.295	0.956	0.705
Maryout 5 (P5)	5.3		5.276	5.25	1.0	0.763	0.172	1.067	0.828

MP; Mean Productivity, GMP: Geometric Mean productivity, HM: Harmonic Mean.TOL: Tolerance Index, STI: Stress Tolerance Index, SSI: Stress Susceptibility Index, YI: Yield Index, YSI: Yield Stability Index.

In the M5 generation, mutant lines G1 and G2 recorded high values of MP, GMP, HM, YI, and YSI compared with their mother cultivars. Mutant line G3 recorded high values of MP, GMP, HM, and YSI, whereas mutant line G4 recorded high values of MP, GMP, HM, and STI. Mutant line G10 recorded high values of HM, STI, YI, and YSI.

### Water productivity (WP kg/m^3^)

Table 10 revealed significant differences (*p* ≤ 0.05) among the selected mutant lines and their mother cultivars for water productivity (WP) in the M3 and M4 generations, whereas differences in the M5 generation were non-significant.

**Table 10:** Water productivity (WP kg/m^3^) of 12 selected wheat mutant lines and their mother cultivars across the M3, M4, and M5 generations under well-watered (Yp) and limited-irrigation conditions (Ys).

Genotype	M3 (Yp)	M4 (Yp)	M4 (Ys)	M5 (Yp)	M5 (Ys)	Grand mean
G1	1.29	1.37	1.09	1.19	1.14	1.216
G2	1.10	1.33	1.09	1.13	1.11	1.152
G3	1.07	1.31	0.97	1.11	1.09	1.110
G4	1.05	1.30	0.93	0.96	1.11	1.070
G5	1.02	1.30	0.95	1.01	1.09	1.074
G6	1.01	1.28	0.88	1.02	0.96	1.030
G7	0.99	1.25	0.86	1.14	0.93	1.034
G8	0.99	1.24	0.85	1.07	1.02	1.034
G9	0.98	1.23	0.81	0.96	0.91	0.978
G10	0.96	1.20	0.79	1.2	1.2	1.070
G11	0.96	1.18	0.78	1.19	1.05	1.032
G12	0.95	1.13	0.75	1.25	1.09	1.034
The mother cultivars						
Sakha 93 (P1)	0.91	0.92	0.58	0.94	0.93	0.856
Sids 13 (P2)	0.92	0.95	0.71	0.86	0.96	0.880
Giza 168 (P3)	0.89	0.86	0.62	0.92	1.08	0.874
Gemmeiza-9 (P4)	0.95	0.88	0.73	0.91	0.96	0.886
Maryout 5 (P5)	0.83	0.91	0.76	0.86	1.07	0.886
SL _0.05_	0.014*	0.017*	0.005*	0.011^NS^	0.007^NS^	–
CV%	9.5	10.2	8.7	11.6	10.0	–
LSD _0.05_	0.10	0.07	0.012	0.30	0.13	–

SL_0.05_: Significant level at 0.05,*: significant, NS: non-significant, CV%: coefficient of variation, LSD_0.05_: Least Significant Difference.

Across generations, all mutant lines consistently showed higher grand mean water productivity (WP) values than their mother cultivars under well-watered and limited-irrigation conditions. However, in the M5 generation, two mother cultivars (P3 and P5) exhibited higher WP values than mutant lines G6, G7 and G9. Based on the grand mean, mutant line G1 recorded the highest overall WP (1.216 kg m^−3^), followed by G2 (1.152 kg m^−3^) and G3 (1.110 kg m^−3^). In contrast, the mother cultivars showed a lower WP grand mean, ranged from 0.856 for P1 to 0.886 kg m^−3^ for P4 and P5.

### Multiple selection criteria

**Fig. 8:**
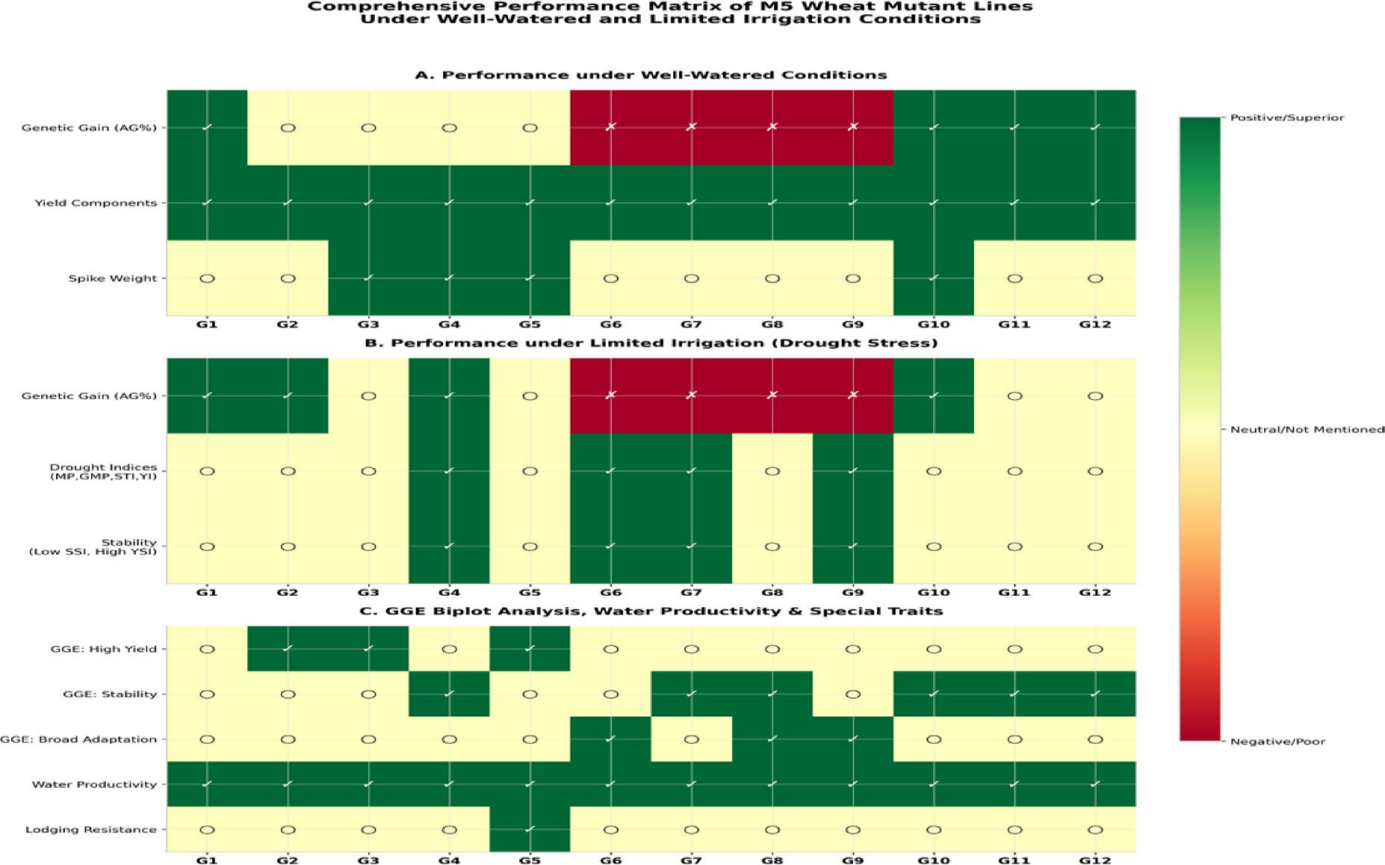
comprehensive performance matrix of selected M5 wheat mutant lines under both conditions based on multiple selection criteria. Green color presented superior performance, yellow indicated average performance, and red indicated poor performance.

Based on **multiple selection criteria**, mutant lines G4, G10, G1, G2, and G5 were detected as the most promising mutant lines for wheat breeding programs targeting high grain yield, drought tolerance, and improved water productivity under both well-watered and limited-irrigation conditions (Fig. 8).

## Discussion

Water scarcity is one of the most critical problems threatening the sustainability of bread wheat production in Egypt. Consequently, improving water productivity through the development of cultivars better adapted to both well-watered and limited-irrigation conditions has become a primary objective of wheat breeding programs^47^. Significant genetic variation (*p* ≤ 0.05) detected among mutant lines across generations under irrigation regimes confirmed the effectiveness of mutagenesis in generating novel genetic variation for the studied traits. Moreover, the significant genotype x irrigation regime interaction indicated differential responses of mutant lines to water availability, reflecting wide variation in adaptive mechanisms and providing opportunities for selecting high-yielding and water-stress-tolerant mutant lines in subsequent generations. Under well-watered conditions, twelve mutant lines surpassed their mother cultivars in key grain yield components, including spike weight, 1000-grain weight, and spikes m^−2^. This superiority was attributed to enhancements in spikes m^−2^, spike weight, and grains per spike, which were major determinants of final grain yield in bread wheat. Enhancement in these traits may have enhanced assimilate accumulation and sink capacity, leading to more efficient utilization of photosynthesis during grain development^48,49^. Mutant line G5 exhibited a lodging-resistant dwarf phenotype characterized by reduced plant height while maintaining acceptable grain yield under both conditions in the M5 generation, making it a valuable donor parent for enhancing lodging resistance in bread wheat^50,51^. This mutant line recorded high yield performance according to GGE biplot, as a dual-purpose mutant line (high yield and lodging resistance). Under limited-irrigation conditions, the same mutant lines maintained a relatively higher grain yield compared with their mother cultivars, suggesting better adaptation to water stress by producing higher spikes m^−2^, heavier spike weight, and higher grains per spike. In contrast, the reduction in grain yield noted in some mutant lines under limited-irrigation conditions highlighted the significant influence of genotype × irrigation regimes on grain yield expression under water-stress conditions. These findings suggested that mutation induction was effective in creating exploitable genetic variability that resulted in improved agronomic performance^18,19^. The estimates of actual genetic gain (AG%) indicated a strong environmental influence on the expression of the studied traits.

Under well-watered conditions, the highest gains were recorded by mutant lines G12, G10, G11, and G1, reflecting the effectiveness of phenotypic selection across generations in improving grain yield potential. Under limited-irrigation conditions, several mutant lines showed reduced gains, indicating susceptibility to limited-irrigation conditions. However, mutant lines G10, G1, G2, and G4 maintained positive gains, suggesting relative tolerance to water stress. The reduction in gain in mutant lines such as G6, G7, G9, and G8 may be due to strong genotype × irrigation interaction and stress sensitivity. Mutant lines G4, G10 and G1 emerged as the most promising lines because of their consistent performance under both conditions, suggesting that these lines have agronomic traits associated with stress resilience. Similar findings were recorded by Abdallah and Mahdy^52^ and El-Hariri and Ramadan^53^, who illustrated the effectiveness of phenotypic selection in combining high yield and drought tolerance in bread wheat.

High broad-sense heritability estimates coupled with moderate-to-high genetic advance (GA%) values indicated that additive gene action predominantly controlled the inheritance of the studied traits, thereby enhancing selection efficiency. These findings justified early-generation selection for detecting promising mutant lines (G1-G12) under both conditions. The observed reduction in genetic variability, accompanied by increased heritability and moderate genetic advance in the M4 and M5 generations, suggested that selection was effective in fixing favorable alleles and enhancing genetic uniformity among the promising mutant lines^23–25^. Correlation coefficients revealed that longer spikes may provide greater physical space for grain development by increasing sink capacity, ultimately contributing to higher final grain yield. In addition, phenotypic selection for higher spikes m^−2^, leading to increased tillering ability and spike fertility, may serve as robust selection criteria for improving grain yield potential^54,55^.

Drought tolerance indices successfully selected mutant lines with high grain yield under both well-watered and limited-irrigation conditions. According to these indices, mutant lines G1 and G2 were selected as valuable genetic resources combining high grain yield with high stability, as confirmed by higher mean productivity, geometric mean productivity, harmonic mean (HM), yield index and yield stability index values compared with their mother cultivars. In addition, mutant lines G3, G4, and G10 were selected as valuable genetic resources based on multiple drought indices, which was desirable for developing widely adapted wheat cultivars. The yield stability index improved markedly from the M3 to the M4 generation for G3 (0.38 → 0.78) and G4 (0.39 → 0.95), indicating transgenerational selection gains in yield across generations. These enhancements were maintained in the M5 generation, suggesting that advanced generations may harbor genetic recombination connected with enhanced stress adaptation. The reduction in genetic variability and GCV in advanced generations may limit further genetic progress. Drought indices have been widely applied for identifying drought-tolerant wheat cultivars under different irrigation regimes^56,57^.

The GGE biplot analysis captured the majority of the total variation and clearly separated the mutant lines from their mother cultivars under both conditions, indicating substantial genetic variability generated through mutagenesis. The GGE biplot analysis identified five mutant lines (G2, G3, G4, G5, and G6) with superior yield performance. Four mutant lines (G4, G7, G10, and G11) were positioned closer to the origin of the biplot, indicating greater stability across environments due to minimal genotype x environment interaction effects, enabling their yield potential to be fully expressed^30–32^.

Estimating water productivity (WP) is a key indicator of drought tolerance, reflecting the plant’s ability to convert available water into final yield. Mutagenesis generated significant variance (*p* ≤ 0.05) among genotypes for water productivity (WP) across generations under both conditions. Based on mean performance, all selected mutant lines exhibited higher WP values than their mother cultivars, as indicated by mean performance comparison. This finding indicated that mutagenesis contributed to the enhanced ability of twelve selected mutant lines to produce high grain yield per unit of added water under both conditions compared to their mother cultivars. The improvements in WP were associated with improved YSI, indicating enhanced adaptation to limited-irrigation conditions. Similar results were reported^58,59^.

Although the current study showed significant genetic variability and selected promising mutant lines under both conditions, it was conducted under particular environments. Therefore, further multi-environment and multi-year evaluations are prerequisite to emphasize the stability and adaptability of promising mutant lines. In addition, molecular characterization would supply a deeper look at the genetic basis of the observed variability and stress tolerance.

## Conclusion

The results of this study demonstrated the effectiveness of mutagenesis in generating novel genetic variation for grain yield, water stress tolerance and water productivity in bread wheat under both well-watered and limited-irrigation conditions. This was confirmed by significant differences among mutant lines across generations under both conditions. Across the M3, M4, and M5 generations, several mutant lines surpassed their mother cultivars in key grain yield components, indicating the potential of induced mutations for improving bread wheat productivity under both conditions. The estimates of genetic parameters indicated the predominance of additive gene action in controlling the studied traits, which increased the effectiveness of phenotypic selection for grain yield components. Mutant lines G4, G10, G1, G2, and G5 were selected as the most promising lines based on grain yield performance, water stress tolerance, and water productivity. Additionally, mutant line G5 was characterized by reduced plant height across generations, representing a potential genetic resource for enhancing lodging resistance. However, the current study was conducted under specific environmental conditions and seasons, and further multi-location and multi-year evaluations are required to confirm the stability of the selected mutant lines across different environments. Overall, these mutant lines could serve as valuable genetic resources for wheat breeding programs aimed at improving climate resilience and sustainable wheat production in water-limited environments.

## Data Availability

The datasets generated and analyzed during the current study are available from the corresponding author on reasonable request. All phenotypic, yield, and water productivity data of the wheat mutant lines across M3–M5 generations under both well-watered and limited irrigation conditions are included in these datasets.
